# Destination Restaurants’ Practices and the Production of Locality: The Case of Michelin Restaurants in China

**DOI:** 10.3390/foods13121838

**Published:** 2024-06-12

**Authors:** Yuying Huang, C. Michael Hall, Ning (Chris) Chen

**Affiliations:** 1 Department of Management, Marketing and Tourism, University of Canterbury, Christchurch 8041, New Zealand; yuying.huang@pg.canterbury.ac.nz (Y.H.);; 2 Geography Research Unit, University of Oulu, 90014 Oulu, Finland; 3 Department of Service Studies, Lund University, 221 00 Helsingborg, Sweden; 4 Department of Marketing and Tourism, Linnaeus University, 352 52 Kalmar, Sweden; 5 School of Hospitality, Tourism and Events and Centre for Research and Innovation in Tourism (CRIT), Taylor’s University, Subang Jaya 47500, Malaysia; 6 School of Culture and Tourism, Ningxia University, Yinchuan 750014, China; 7 Department of Geography, Kyung Hee University, Seoul 02447, Republic of Korea

**Keywords:** food tourism, destination restaurant, Michelin Guide, locality, local food, gastronomy tourism

## Abstract

Dining plays a pivotal role in the travel experience, with numerous studies identifying the significant impacts of restaurant attributes on tourists’ destination experiences and their sense of place. The identified attributes include the origin of food produce, menu design, the physical and social servicescape, and restaurant reputation, all of which have the potential to enhance customers’ sense of place. Therefore, based on theories of the production of locality, this study explores how destination restaurants “put place on the plate” and identifies how destination restaurants promote place. Semi-structured interviews were conducted with the representatives of seventeen Michelin (one star, two stars, three stars, and Bib Gourmand)-awarded restaurants across Mainland China. The results reveal three primary strategies employed by destination restaurants in promoting place: forging partnerships with the local community to produce, present, and reproduce localities; leveraging local knowledge embedded in the local produce, recipes, cooking techniques, and local culture; and practicing translocality to introduce a regional cuisine to diverse and cosmopolitan consumers. This research provides a comprehensive understanding of the way in which notions of locality and place are used by destination restaurants and the way in which this may promote not only restaurants but also regional culinary cultures and destination attractiveness.

## 1. Introduction

Tourism is a major economic and social force in (re)producing places and cultures [1]. Food has long been regarded as a key attraction for tourists, and local food and local culinary culture are examples of discursive signifiers of tourist mobility [2]. Destination restaurants play a significant role in attracting tourists by offering exclusive culinary experiences. Within this perspective, some destination restaurants may be ‘ambassadors’ that promote place and locality [3,4], and some of them, namely ‘terroir restaurants’, have indeed gained market recognition and received many honours [5,6,7]. As a result, destination restaurants could be considered as a representation of the local culture, a counter to the homogenisation of cuisine by globalising forces [8], and a source of destination marketing [9]. Locality promoted in destination restaurants is therefore potentially a significant form of local cultural capital that strengthens a sense of place, place and product differentiation, and place branding in a globalised world [10,11,12]. This is also related to the restaurant’s authenticity, sustainability, and destination branding [5,7,13]. However, globalisation processes have made traditionally distant geographies less socially and culturally relevant due to the introduction of global industrialised food systems such as fast-food chains [12,14], which is paradoxical to the food trend of promoting locality in destination restaurants. The use of place and locality by restaurants to promote themselves or their menus is by no means ubiquitous [7]. Therefore, how destination restaurants can achieve global recognition while promoting locality is a topic worthy of discussion [15].

The production of locality highlights the social mechanisms to obtain local knowledge and produce local subjects by production, representation, and reproduction in local communities, as well as emphasises the phenomenon of translocality contributed by the mobility of populations [16]. The theory has been widely employed in various social science disciplines to analyse, for example, festivals [17], online tourism peer-to-peer platforms [18], musical practices [19,20,21], literary geography [11], immigrants governance [22,23], and social class [24]. All these studies employed the production of locality to analysis a ‘placemaking’ process and the interactions between local community and place. Given that creating a destination restaurant also involves placemaking [25], this article applies the production of locality to analyse the destination restaurant’s practices that have potential to promote place.

This study explores how destination restaurants promote place by taking a constructivist approach. To do this, the research objectives are twofold: (1) to identify what restaurant’s practices have potential to represent locality; (2) to explore how these restaurant’s practices could promote local place. The focus of this study is on Michelin restaurants in China, which could be recognised as the most high-profile restaurants in China considering the global visibility of the Michelin Guide [26]. Meanwhile, the unique history, background, architecture, and culture outlooks of China also nurture distinguished culinary cultures and become a strong justification for the preservation of the locality [27,28]. This article advances our knowledge in the nexus of people–food–place by applying the production of locality. Therefore, this pioneering study provides new understandings of the way in which notions of locality and place are used by destination restaurants in China and how this may promote not only restaurants but also culinary culture and destination attractiveness. The findings also provide recommendations for restauranteurs and destination planners to reinforce place attractiveness and competitiveness.

In the next sections, this article first introduces and conceptualises the production of locality and destination restaurants and then reviews restaurants’ practices that have potential to promote place; then, this article demonstrates the method, analysis, and findings of the study. Finally, the conclusion and implications are presented.

## 2. Literature Review

### 2.1. Destination Restaurants and Place

Food has become an important attraction to enhance destination image and is an essential element of the tourist experience. Food tourism is defined as “visitation to primary and secondary food producers, food festivals, restaurants and specific locations for which food tasting and/or experiencing the attributes of specialist food production region are the primary motivating factor for travel” [25] (p. 10). The discourses of food tourism are shaped by five driving forces [29]: (1) political capital, where food is an element of a nation’s culture and identity, along with its history, symbols, myths, and discourses; (2) a visionary state, where tourists desire to be; (3) what it means to be a foodie, who regards food as the source of all moods and all sensations, a focus for socialising and a means for simultaneous enriching experiences, expressing personal identities and adding to quality of life; (4) the drive for affluence and exclusivity with expensive, rare, and exotic food; and (5) the diversity of experiences through sampling a wide range of novel and familiar experiences.

As gastronomy has become a distinctive tourism product, restaurants play a significant role in tourism because tourists, like anyone, have to eat. Dining in local restaurants gives visitors clues about the local way of living, manners, geography, economy, and related cues [30]. In the supply chain of food services, restaurants have opportunities for putting food into the public spotlight by demonstrating culinary possibilities and introducing them to the local economy [31]. Perhaps not surprisingly therefore, restaurants could be considered as agents of culinary and cultural change [32]. From the perspectives of customers, restaurants can also directly promote their localities, not only in terms of food but also place, people, and cultural contexts [33,34]. The consistency with food cultures’ history, ethos, and aesthetics is fundamental to the endurance of a sense of connection felt by community members and to the sustainability of that food culture [35]. Based on the definition of food tourism, this study regards restaurants that could play as a role in attracting domestic and/or international tourists travelling to the destination as destination restaurants. However, not just any visit to a restaurant is considered as food tourism; the destination choice of the tourist must be shaped by a special interest in culinary attributes, gastronomy, or the cuisine. In addition, local residents should also be able to eat at destination restaurants.

### 2.2. The Production of Locality

There is a long-standing concern in the social sciences with the “local”, which is produced by places and how one place is distinguished from others [16,36]. The notion of locality is different from place; places do not represent a permanent natural datum, but rather a context generated by the actions through which different social actors build their relational systems in space and do so by using various strategies for identifying and accordingly mapping the identity elements that characterise each locality [16]. Locality is defined in relation to the concept of “neighbourhood” “as a complex phenomenological quality, constituted by a series of links between the sense of social immediacy, the technologies of interactivity, and the relativity of contexts” [16] (p. 178). Appadurai discerned locality as “a dimension or value”, while defining the neighbourhood as “existing social forms”. In the restaurant context, locality is viewed as largely geographical understandings of products ‘made’ in the region, as well as more complex understandings based upon economic, social, and cultural factors [3].

The production of locality indicates the social mechanisms used to (re)produce local subjects by production, representation, and reproduction in neighbourhoods [16]. This process involves three elements: local communities, local knowledge, and translocality. *Local communities*, or “neighbourhoods”, both are contexts and at the same time require and produce contexts; “they provide the frame or setting within which various kinds of human action (productive, reproductive, interpretive, performative) can be initiated and conducted meaningfully” [16]. Within this perspective, destination restaurants can thus be viewed as a mechanism socially, materially, and environmentally grounded in the local context that then engage local subjects in the social activities to the creation of local contexts. *Local knowledge* is substantially about producing reliably local subjects as well as about producing reliably local neighbourhoods within which such subjects can be recognised and organised [16,37]. In destination restaurants, local knowledge might contain the food and eating habits in the forms of local ingredients (plants and animals), cooking techniques, and gastronomic movement [38,39,40]. *Translocality* emphases a mobile situatedness of the individual, which is a mass-mediated decoupling of identity and territory in which experiences and social actions regarding taste, pleasure, and politics can unexpectedly converge and intersect [16,18]. Scholars argued that translocality views such processes and identities as place-based rather than exclusively mobile, uprooted, or ‘travelling’ [41]. However, in the globalisation era, food cultures constantly move with immigration, labour mobility, and translocal production shifting from their source regions to others [42]. 

The production of locality is employed as the theoretical support in this study for three reasons. First, destination restaurants could be regarded as the epitome of local community, which could (re)produce and represent local subjects in terms of culinary culture and food system. Second, the food culture displayed in destination restaurants would be influenced by globalising forces, yet these do not always overwhelm local distinctions and the international and domestic co-exist and coalesce [43], which could be expounded by locality and translocality. Third, the theory also points towards the term ‘production’, referring to making and creating, as well as to the not-yet-existing, and thus also to the future [17], considering the role of a destination restaurant in both creating people–place ties and opening this place to the world.

### 2.3. Restaurant Practices That Introduce Locality

Previous studies have found that destination restaurants could demonstrate locality in various ways during the processes of procurement, preparation, presentation, and promotion [7,34]. This study further introduces four main practices related to promote locality: sourcing local foods during procurement [44,45,46], introducing local culinary culture in the menu during preparation [40,47,48], demonstrating the restaurant servicescape with locality elements during presentation [49,50,51], and establishing restaurant reputation during promotion [52,53,54].

*Local food* is defined based on distance, political boundaries, and speciality criteria, such as geographical designations and indications [55]. Sourcing local foods is a significant practice of locality, which are usually fresher, healthier, and more sustainable for the environment, and produces values such as originality, newness, and locality, as well as authenticity and uniqueness for food tourists [44]. In considering socio-cultural sustainability, the maintenance of local foodways and production is crucial for preserving local traditions and increasing local communities’ pride; in return, local communities should also be involved in creating and preserving local dishes [56]. Proximity is the key to understanding local foods, including geographical proximity; relational proximity; and social, economic, and environmental proximity [34]. Scholars reviewed two decades of research on local food systems and generalised eight benefits of LFS within the perspectives of consumers, farmers, community, and environment [57]: (1) increasing consumers’ access to fresh and healthy food; (2) strengthening consumer willingness to pay more for local over non-local foods; (3) enhancing farmers’ sense of social recognition; (4) economically benefiting farmers; (5) increasing local community ties; (6) benefiting the local economy; (7) fostering environmentally friendly production practices; and (8) helping mitigate climate change. Nevertheless, a number of perceived barriers to restaurant procurement of local foods have been identified, including a lack of knowledge, inconvenient ordering and delivery times, limited availability of products, variable costs, packaging and handling, and inadequate distribution systems [58,59]. 

A *menu* is an essential advertisement in consumers’ hands before dining in the restaurant [60], which provides an early impression of a restaurant, as consumers rely on its representation to make consumption decisions [61]. Menu design is a process connected to graphic design, marketing strategy, cost management, co-branding with food tourism, and nutrition that provides information about the food service in terms of menu item position, description, place of origin, and characteristics [62,63]. Scholars employed document review methods in restaurants’ menus and identified five motivational factors for visitors to consume local food: quality of taste, authentic experience, rural development, health concern, and knowledge [64]. Expanding the focus on the meal producer and consumer, the menu could further be understood within the wider context of tourism and food systems [2]. An informative menu with food details could also indicate traditional food practices and culinary heritage and elements of the food supply chain to customers, such as local ingredients and traditional cooking techniques [26,33]. While food with a local origin is crucial in menu design, chefs acknowledged the difficulty of building seasonal and sustainable supply chains and the impact of global influences on the restaurant experiences [47]. It is possible that not disclosing menu details can also be regarded as an alternative marketing strategy to spark further interest with consumers and suggest freshness and daily determination of what is served [65]. 

*Servicescape* refers to “the ability of the physical surroundings to facilitate achievement of (internal) organisational as well as (external) marketing goals” [66]. Previous studies have proposed various sub-dimensions to evaluate the physical servicescape of restaurants, which could be classified for functionality and aesthetics [67,68,69], such as the restaurant’s building, name, tableware, and decoration. Moreover, social servicescape highlights the presence of others in the service environment that could also influence customer experience [70]. In the restaurant context, the social servicescape usually refers to the interaction between customers and staff and the interaction between customers [71], for example, when the chef/waitstaff verbally describes a menu’s ingredients, producers, and cooking methods to customers [33]. However, the e-servicescape on a restaurant’s website or social media has become increasingly important, especially in the post-pandemic era [72]. Restaurant-based e-services can be classified into informational, transactional, and interactive services [73,74], which induce customer’s perceptions of e-servicescape and influence customer experiences. These restaurant servicescape elements can reinforce place information, offering an opportunity for customers to learn about local culture and foods and the host destination [50,71,75].

*Reputation* reflects a mixture of reliability, admiration, benevolence, respect, and confidence of an organisation [76]. In the service sector, reputation is particularly important, given services’ intangible character and the difficulty in evaluating its quality without having experienced it [77]. Customers can assess restaurants even if they have only heard about the experiences of other customers or about the restaurant via media or other information sources [78]. The consistency of a restaurant’s reputation and service quality could contribute to a customer’s positive attitude toward the brand [79]. Destination restaurants could promote locality through their reputation. For example, restaurant stories and culinary concepts have gone around the world and influenced the whole foodservice industry through approaches such as restaurant awards [80], celebrity chefs [81], community outreach [34], media communication [82], and food events [30]. With the boom in food media, there is increased importance in using local chefs over non-local or celebrity chefs because of this new desire for authenticity and local cuisine [83]. In addition, many restaurants see themselves as part of a local community rooted in their regions, relying on local products and representing and shaping the place [2]. Therefore, they are more likely to take an active role in encouraging improved sustainable behaviours in their community, including community-based gardens, cooking events, charity work, and encouraging sustainable forms of eating as well as different forms of community-based activism [34].

Overall, destination restaurants could provide tremendous opportunities to put localities into the public spotlight through various practices in the categories local food, menu, servicescape, and reputation. Their high profile may also have profound impacts on their customers, the restaurant industry, the food system, and the place in which they are located. Within this perspective, locality comprises not only food but also, place, and cultural context conveying national and global elements [33]. The nexus of people–food–place is dynamic [84]; this study thereby argues that the localities could be produced, presented, and reproduced in destination restaurants based on the production of locality. Previous studies investigating locality in restaurants were usually conducted through case studies and from Western perspectives [33,85,86,87]. In contrast, this research is undertaken via interviews and in an Asian context. However, it is noteworthy that the notion of locality (re)produced and presented in destination restaurants has not been questioned, especially in an oriental context.

## 3. Methods

### 3.1. Research Sample

This study examines restaurants that have been awarded Michelin Guide distinctions as the sample of destination restaurants, including (1) three-star restaurants, which “provide exceptional cuisine, worth a special journey”; (2) two-star restaurants, which have “excellent cooking, worth a detour”; (3) one-star restaurants, which are “very good restaurants in its category”; and (4) Bib Gourmand restaurants, which “offer exceptionally good food at moderate prices” [88]. The Michelin Guide is regarded as a tastemaker of contemporary food and restaurant culture, wielding both symbolic and material power in the global restaurant industry [7] and informs decisions on destination restaurant visitation [89]. Some countries’ governments have also cooperated with Michelin to create official Michelin Guides to promote local culinary culture [90]. 

This study uses the Michelin restaurants in Mainland China as the research sample. With a long history of culinary culture development and a vast and varied gastronomic landscape that reflect different geographical regions [91]. Under the influence of globalisation, non-Chinese food cultures have also had profound impacts on traditional Chinese cuisine during the past century by introducing global fine-dining restaurant brands [28]. Considering the large consumer market and culinary culture, restaurants in China have begun to attract worldwide attention from the restaurant, hospitality, and tourism industry and have increasingly become incorporated into global assessments of culinary quality [26]. As an emerging food destination, food tourism and destination restaurants in Mainland China are worthy of attention since the current movement and direction of food tourism may have different implications to those in established food destinations, for example, Singapore [92], Thailand [93], Japan [94], Korea [95], Malaysia [96], Indonesia [84], and Vietnam [45]. Although some scholars have explored food tourism in China [97,98,99], they usually focus on a specific region. However, research on destination restaurants in Mainland China that have strong potential to promote locality remains unexplored. 

Although the Michelin Guide in China attracts some controversy, such as the inability of foreign judges to understand elements of the local cuisine and foodways [100], it still presents a key event in the integration of Chinese gastronomy into a globalising culinary field and plays as an effective pull factor for tourists [101]. As of April 2024, the Michelin Guide has explored five cities in Mainland China, including Beijing, Shanghai, Guangzhou, Chengdu, and Hangzhou, and has recognised 240 distinguished restaurants in these cities. The number of restaurants by city and distinction is shown in Table 1.

### 3.2. Research Design, Data Collection, and Analysis

This study employs constructivism as the research paradigm and utilises an interpretive methodology to explore how destination restaurants promote place. The constructivism approach posits the utilisation of multiple realties to elucidate a phenomenon, thereby enabling the subjective understanding for knowledge creation [102]. The interaction between the researcher and respondents elicits individual interpretations of reality or the relationship with the phenomenon, thereby facilitating researcher’s understandings of the phenomenon within the respondents’ social contexts, and the empirical materials are collected from the insider’s view [103]. 

The qualitative data were collected in Mainland China in November 2023. A purposive sampling method was employed, targeting the restaurant representatives of Michelin restaurants in Mainland China, such as founders, chefs, restaurant managers, restaurant consultants, and marketing managers, due to their comprehensive knowledge of their represented restaurants and their in-depth insights about the industry based on working experience. The first author searched the publicly available contact information from identified restaurants’ websites or social media accounts and then invited them to participate, accompanied by an information sheet and a consent form sent via email. With respect to restaurants that do not display their contact information online, such as small shops and street food hawkers, the researcher directly engaged with potential participants by visiting the restaurants, introducing the research, and enquiring on their willingness to participate the interview. 

The sample size was not set in advance; rather, the approach of seeking the point of data saturation was used [104]. A point of data saturation is reached when additional interviews do not lead to additional insights; saturation may occur within the first twelve interviews, although the basic elements for metathemes are often present at as early as six interviews [105]. Such an approach is also widely used in qualitative restaurant research [106,107,108]. For the present study, this point was reached at 17 interviews. As Table 2 illustrates, 17 respondents representing 21 restaurants participated in this study.

Semi-structured interviews were used [109], and a tunnel technique with both central and additional questions was applied to examine how destination restaurants “put place on the plate” and identify how destination restaurants promote place [110]. The first author followed a script of questions based on the literature review, which included the topics local food, menu, servicescape, and reputation. Interviews were conducted in either the respondents’ restaurants or online meeting platforms and lasted, on average, for 45 min. The mother-tongue language, Mandarin, was the main language for interviewing to ensure that the respondents fully understood the questions and allowed questions to be asked to have a better understanding of the answers. The interviews were audio-recorded with the participant’s informed consent. The verbatim interview was transcribed and translated from Chinese to English following a back-translation technique for data analysis [111]. 

An inductive data analysis was conducted to identify how the destination restaurants promote localities by using open coding, axial coding, and selective coding. This study utilised a multiple analysis process to enable concepts and categories to emerge [84]. First, the text data were reviewed and familiarised. Second, initial coding classified the massive amount of data into itemised meaningful concepts. Third, the concepts were classified into categories according to mutual elements in the codes, recurring statements, or opinions in the coded text, metaphors, and analogies and by comparing differences. Fourth, the researchers explored the possible links and associations between the reviewed concepts, sub-categorises, and core categories. To enhance the validity of the research, a cross-check of multiple sources of evidence, such as interview transcripts, documentation, online presence, and photographs, was conducted and is detailed in the Findings and Discussions. To enhance the reliability, direct quotes, a documentation analysis, and information from authoritative media (e.g., the restaurant’s official website, office social media account, and previous interviews reported by other institutions) were used to corroborate the findings as well. 

## 4. Findings and Discussion

The first research objective was to identify what restaurant’s practices have potential to represent locality. By conducting semi-structured interviews, this study further specifies the key practices for restaurants to represent locality. A summary of the analysis results is illustrated in Table 3. According to notions of the production of locality [16], this study further identifies these practices as local community, local knowledge, and translocality and clarifies their roles in promoting local place in Mainland China.

### 4.1. Local Community

Based on the theory of the production of locality, local communities both provide and require contexts to produce locality through production, representation, and reproduction within their communities [16]. This research also identifies the significance of the local community in presenting locality in destination restaurants. Local communities, including local suppliers, local customers, and local teams, are the basis of destination restaurants in Mainland China.

#### 4.1.1. Local Suppliers

Destination restaurants are always keen to search for high-quality ingredients as it is a key factor that influences a restaurant’s quality. All the respondents emphasised the importance of high-quality ingredients. One of the restauranteurs (R12) stated that “our kitchen holds no secrets; our secrets lie within the ingredients”. Based on the results of the interviews, high-quality ingredients mean a fresh, the best, and a stable supply; to achieve these, the restaurants should establish trust with local suppliers. 

Many respondents (R1, R10, R11, and R12) mentioned that they required the ingredients to be delivered to the kitchen from their source in one day or even one night, especially as the restaurants are heavily reliant on river and sea produce. One respondent (R10) highlighted the seafood from Dongshan Island, Fujian Province, and mentioned that local fishers usually start shipping around midnight, with the catch typically retrieved at 2:00 am to 3:00 am, arriving at the dock by four or five; they then rush to catch the seven or eight o’clock flight, ensuring arrival in Shanghai, which is approximated 1200 km away, before lunch time to keep the seafood live; and they repeat this work every day. This process requires seamless cooperation between restaurants, fishers, and local workers for packaging and transport. 

Another respondent (R12) emphasised the trust between him and his suppliers, who are his relatives or childhood friends, to always reserve the best or the rarest fish for him even if someone offers a higher price. With trust established, the local suppliers are then willing to collaborate with the restaurants to make some changes. For example, “chickens and ducks are tactically raised for only 200 days, however, we request them to extend the rearing period, like 250–300 days before supplying them to us” (R11). In return, promoting local food in destination restaurants could also increase their demands and thereafter protect the locality. For example, a respondent (R15) said that their restaurant is the first to use wine lees steamed buns from twenty years ago, procured from a small bun shop.

*“The lady who make them initially refused as no one bought her products, then we said, ‘you just make them for us’. And gradually, we see now, these wine lees steamed buns are already very popular in Hangzhou, and it is only when there has the demand that people will think about inheriting it”*

(R15).

The stable supply of good ingredient is of importance. R6, representing one of the three-star Michelin restaurants in Mainland China, indicated that they only launched five menus in the last decade, so the chef prefers to utilise local products to ensure a stable supply, such as the truffles from Yunan province, which offers a more stable supply compared to those from France or Italy. Within these perspectives, utilising local ingredients could showcase the prominence of local working people within the supply chain and thereafter promote the place, which is consistent with the findings from previous studies [112,113,114].

#### 4.1.2. Local Customers

Most of the studied restaurants prioritise local customers over outsiders, which might be due to the enormous domestic demands in Mainland China [115]. “Normally over 50% of customer were international visitors before the pandemic, […], we’re lucky to get very strong support from the Chinese customers and local experts, especially during the last three years in pandemic, […], our tables are always fully booked” (R6). Destination restaurants usually satisfy the local market in several ways, including using local ingredients, providing local cuisine, and communicating with local customers. A restaurant consultant (R1) indicated that “from a utilitarian perspective, serving local cuisine made with local ingredients in the local area will undoubtedly attract the most customers”, he applies this thought when he designs restaurants. Another manager (R3) said that “when most dishes are already prepared during peak time, he [the chef] comes out and talks with the regular customers, the customers also know him and discuss the flavours with him in local dialect”.

It is interesting that the identified restaurants providing either local or domestic cuisine prioritise local customers. For example, Shanghai is a megacity gathering people from all corners of the globe. A restauranteur (R7) providing authentic Shanghai cuisine always informs their customers that the cuisine is very sweet compared to the other Chinese regional cuisines. From the perspectives of domestic cuisine, the restaurant (R10) has branches into several cities across Mainland China, and they noticed that the customers in Shanghai order more appetisers than others so they provide more options of appetisers on their menu. As for ethnic restaurants, a restaurant manager (R16) indicated that they applied French cooking techniques to local produce that the local customers is familiar with, which also makes their restaurant unique and attractive. As for the reasons for emphasising the role of local customers, scholars have identified that repeat customers are found to be sensitive to quality variations, which strongly influence a restaurant’s reputation and (online) word-of-mouth, while such sensitivity is even accentuated by local customers [116]. Adjusting the restaurant offerings to satisfy the local market has also been identified in other studies [117,118,119].

#### 4.1.3. Local Team

Some restaurants have a long history, spanning hundreds of years, and enjoy distinguished reputations within the industry and local market. For example, a restaurant offering Sichuan cuisine has been inherited by four generations in a family.

*“Their great grandfather opened this restaurant a hundred years ago, both the grandfather and the father grow up in the restaurants and then inherited the restaurant, now the (grand)daughter also aspires to carry forward and extend the restaurant’s legacy, …, many of their colleagues are childhood friend, knowing each other well and sharing the same goals”*

(R17).

When restauranteurs who are not locals were asked why they came and stayed, one respondent (R6) answered “It’s basically about the opportunity here and the support he [the chef] got eventually here. For the first few years, he started to build his own team, and his team is based here. Along the way he got opportunities here, so he stays here”. From these perspectives, destination restaurants also display social responsibility. A respondent (R8) mentioned that their restaurant recruits hundreds of employees, ensuring their well-being and considering their housing and family needs, which are contributions to society. Regarding the relationships between these destination restaurants and their employees, such local communities are the main actors in producing localities for destination restaurants. Similarly, some previous research on restaurants also emphasised the significance of local teams or local employees [38,120,121].

### 4.2. Local Knowledge

The production of locality suggests that local knowledge could be regarded as a record of the spatiotemporal production of locality, which is knowledge of how to produce and reproduce locality under various conditions [16,37]. This study indicates that destination restaurants present local knowledge through their products, recipes, cooking techniques, and local culture, and therefore, these elements could promote locality in different conditions.

#### 4.2.1. Products

Local knowledge could help destination restaurants to understand local produce to distinguish their ingredients’ quality. For example, one respondent (R1) said:

*“I tend to focus on ingredients from the Yangtze River Delta (Jiangsu, Zhejiang, and Shanghai), including those from the East China Sea. You could say it is my comfort zone or the area where I grew up. I am more familiar with this region and lean towards using ingredients from these areas… I might delve into the finer details of regional ingredients. For instance, as I mentioned the eel from the rivers. In the upper reaches of the Qiantang River in Hangzhou, there are actually three segments, and the texture of the eel varies in different river basins. Many connoisseurs can distinguish between them, and the prices also vary greatly”*

(R1).

With their knowledge of the local produce, some restaurants also grow and make food themselves. For example, a restauranteur (R13) indicated that her family has some farmlands on the outskirts of the city so they grow some vegetables and supply them to the restaurant; she also emphasised that they follow seasonal and organic agriculture principles. Numerous studies have examined the motivation to employ local foods in restaurants [33,119,122], but this study further emphasises that the familiarity of local products and the controllability of local food quality are also significant motivations for restaurants to utilise local products.

#### 4.2.2. Recipes

Many respondents (R1, R2, R4, R5, R11, R12, R14, and R15) indicated that their restaurants procure food ingredients and design a menu guided by the Chinese 24 Solar Terms because Confucian culture advocates for refraining from eating that which is not in season. The recipes also refer to local culinary heritage. For instance, one respondent (R3) highlighted “the Chef Studio”, organised by their group, which unearths forgotten dishes. They retrieve recipes and then conduct experiments. By remaking these dishes, they excavate and preserve culinary heritage. 

Many restaurants seek to improve traditional recipes. A restauranteur (R11) indicated that their traditional dishes no longer use shark fin for environmental reasons. Low-salt, low-sugar, and low-oil food trends also challenge Shanghai cuisine, which traditionally involves rich sauces and oil. Thus, the local restaurants made some modifications; they utilise rock sugar or maltose instead of traditional granulated sugar (R3, R7, and R8). Therefore, this study argues that leveraging the local knowledge embedded in traditional recipes improves them over time and is more important than simply inheriting them unchanged. The theoretical framework of recipes is constructivist, which rests on three factors: the expertise required on the part of the cook; authenticity (in turn, based on the fit and approval rating of any purported rendering); and the open-ended character of recipes [123]. Therefore, inheriting and improving traditional recipes could be considered as a means to promote local knowledge in destination restaurants, which has also been suggested by previous studies [124,125,126].

#### 4.2.3. Cooking Techniques

The identified destination restaurants tend to apply the cooking techniques from where their cuisine originated to cook local ingredients. However, the French restaurant represented by R16 utilises classic French cooking techniques to cook local food ingredients. The restaurant represented by R1 highlights the frying cooking technique, which is widely used in all types of Chinese cuisine. The chef’s mastery of frying techniques significantly influences their reputation within the restaurant industry and adds to the restaurant’s prestige. This restaurant features an open kitchen where chefs demonstrate to customers how the dishes are fried, as a “front-of-house Chinese cuisine.” The restaurant represented by R3 refers to their culinary craftsmanship of the “oil-blasted shrimp” as an intangible cultural heritage, which cooks local river shrimp within 18 s. In return, presenting these traditional cooking techniques is also crucial for conserving the associated local knowledge [26,39,127].

#### 4.2.4. Local Culture

**Figure 1:**
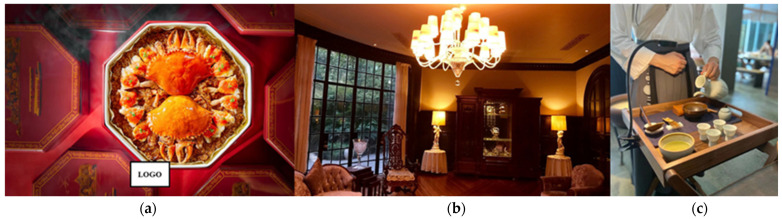
Destination restaurants presenting local culture: (**a**) tableware, image from a respondent (R11); (**b**) decoration and architecture, image from the restaurant’s TripAdvisor; (**c**) service of a tea-pouring ceremony, image from a respondent (R14).

Destination restaurants also present the localities in their tableware, decoration, architecture, and service and in the name of their restaurant. Figure 1a demonstrates a plate designed by a restauranteur (R11); the octagonal design is inspired by the local architectural feature, and a local famous tourist attraction is depicted on the lid. A respondent (R8) mentioned that their restaurants are all located in local traditional buildings, and they also collect some ornaments from residents to decorate their restaurants, such as some lamps, sofas, and even window frames and floor tiles, which are shown in Figure 1b. In Figure 1c, the restaurant represented by R14 performs the tea-pouring ceremony from the Song Dynasty for each table of guests. The restaurant represented by R10 explained that the restaurant’s name and philosophy were derived from the name of a local river, making it more traceable and rooted in the region. Similar findings were also identified in previous research on restaurants [26,90,128].

### 4.3. Translocality

The production of locality highlights the translocal phenomena in superdiverse societies due to a mobile situatedness of the individual [16,18]. Food culture also constantly moves with immigration and translocal production from indigenous regions and changes through the process of collision and fusion with others [42]. This study further explores the translocality presented by destination restaurants in two aspects: menu formulation and introducing indigeneity. 

#### 4.3.1. Menu Formulation 

One method of menu formulation is combining indigenous cooking techniques and local products to satisfy the local market, as discussed in the previous sections. Many respondents also highlight the term “Shou Zheng Chuang Xin” [守正创新], which means adhering to tradition while innovating. But, in this process, the question of how to balance the percentages of locality and indigeneity arises. This study also found that the discussion on balancing innovation and tradition in menu formulation never stops [83,126,129]. Respondent (R10) provided a good answer to this:

*“I believe human acceptance is based on familiarity. I need to build up psychologically before trying something new. It must be based on something familiar, and then gradually incorporate subtle changes. There is a book by Songwei Li named ‘5% Changes’, your changes may be controlled within 5%, which is a reasonable range”*

(R10).

#### 4.3.2. Introducing Indigeneity

On the other hand, it is also important for restaurants to introduce themselves well. For instance, respondent R3 can accurately translate the dishes’ names from Chinese into English only after carefully studying the ingredients, cooking techniques, seasonings, and utensils used for making the dishes. Respondent (R11) and his restaurant are among the first to brand Fujian cuisine nationwide; “our restaurant in Shanghai lost over a million yuan in the first month; luckily, I have confidence in our quality and in Fujian ingredients. … The restaurant gradually gained popularity thanks to positive word-of-mouth from diners”. He also indicated that years ago, Fujian cuisine restaurants were unconfident about calling themselves “Fujian cuisine” while using terms like seafood restaurant or Hokkien–Cantonese restaurant, but in recent years, more restaurants have proudly proclaimed themselves as Fujian cuisine restaurants, which is a positive development. In short, this study argues that translocality within destination restaurants could be successful only when merging locality and indigeneity based on the local community and local knowledge. Previous research also provides similar insights that the dynamics of the formation of a new epistemic movement depend on the form and nature of the interactions between ‘local buzz’ and ‘global pipelines’ and on the capacity of the originating community to develop and diffuse new rules and ‘episteme’ on a global scale while consolidating them locally [38,130,131].

## 5. Conclusions and Implications

Food is a key attraction for tourists, and locality has become a significant form of local cultural capital that strengthens a sense of place, place and product differentiation, and place branding for food in a globalised world. In some cases, this may extend to restaurant menus, promotion, and positioning, including with respect to so-called ‘destination restaurants’. These destination restaurants are an important component of food tourism because of the significant role they play in attracting tourists by offering exclusive culinary experiences. Drawing upon the production of locality, this study identifies the locality-based practices within destination restaurants that have potential to promote place. By conducting 17 semi-structured interviews with Michelin restaurants’ representatives in Mainland China, the results reveal three primary strategies employed by destination restaurants in promoting place: forging partnerships with the local community to produce, present, and reproduce localities; leveraging local knowledge embedded in the local produce, recipes, cooking techniques, and local culture; and practicing translocality to introduce a regional cuisine to diverse and cosmopolitan consumers. 

This study implies both theoretical and practical contributions. For theoretical contributions, this study comprehensively understands the way in which notions of locality and place are used by destination restaurants and the way in which this may promote not only restaurants but also culinary culture and destination attractiveness. Considering destination restaurants as an attraction of the local place, this study provides insights into how destination restaurants contribute to the local economy, environment, community, and culinary culture, which would further be beneficial to the destinations in terms of place reputation, place branding, and placemaking [9,10,132]. This study also implies practical contributions for restaurant managers and destination planners. Considering the high level of competition between destinations [133], this study provides potential destination competitiveness frameworks to discover new business opportunities and strategies to attract tourists, including suggestions for improving destination restaurants’ online presence, inheriting local culinary culture, and marketing local culinary culture as a part of destination offerings that could further enhance the destination attractiveness.

In terms of the limitations and future studies, first, this study only interviewed restaurant’s representatives in Mainland China. Future studies could explore the locality promoted within destination restaurants in other areas. Second, from the findings, the researchers assume that the locality dimension of destination restaurants might be varied by the type of restaurant (e.g., terroir restaurants, ethnic restaurants, and family restaurants). Thus, future studies are encouraged to provide a greater variety of types of restaurants in researching similar topics. Third, given that this study also highlights the significance of suppliers and customers in perceiving locality in destination restaurants, future research would be fruitful in researching suppliers’ and customers’ perceptions of locality.

## Figures and Tables

**Table 1:** The Michelin Guide restaurants in Mainland China (*n* = 240).

City	Three-Star	Two-Star	One-Star	Bib Gourmand	Total
Beijing	3	2	27	20	52
Shanghai	2	8	41	26	77
Guangzhou	0	3	15	42	60
Chengdu	0	2	11	20	33
Hangzhou	0	0	6	12	18
Total	5	15	100	120	240

**Table 2:** Participant demographics (*n* = 17).

Participant ID	Role	Working Experience	Michelin Distinction	City	Cuisine Type	Price *
R1	Consultant	10 years +	One-star	Shanghai	Cantonese	¥¥¥¥
R2	Manager	10 years +	Two-star	Shanghai	Cantonese	¥¥¥
R3	Communication	15 years +	One-star	Shanghai	Shanghainese	¥¥
R4	Chef	15 years +	Bib Gourmand	Shanghai	Shanghainese	¥¥
R5	Manager	5 years +	One-star	Shanghai	Cantonese	¥¥
R6	Communication	20 years +	Three-star	Shanghai	Innovative	¥¥¥¥
			Bib Gourmand	Shanghai	French	¥¥
R7	Founder	20 years +	Bib Gourmand	Shanghai	Shanghainese	¥¥
R8	Communication	20 years +	One-star	Shanghai	Shanghainese	¥¥¥¥
			One-star	Shanghai	Shanghainese	¥¥¥
			One-star	Shanghai	Shanghainese	¥¥¥
			One-star	Shanghai	Vegetarian	¥¥¥¥
R9	Manager	5 years +	One-star	Shanghai, Chengdu	Sichuan	¥¥¥¥
R10	Communication	5 years +	Bib Gourmand	Shanghai	Fujian	¥¥
R11	Founder	20 years +	One-star	Shanghai	Fujian	¥¥¥
R12	Founder/Chef	20 years +	Bib Gourmand	Hangzhou	Zhejiang	¥¥
R13	Manager	15 years +	Bib Gourmand	Hangzhou	Hang Zhou	¥¥
R14	Manager	10 years +	One-star	Hangzhou	Zhejiang	¥¥¥
R15	Manager	15 years +	One-star	Hangzhou	Zhejiang	¥¥¥
R16	Manager	5 years +	One-star	Chengdu	French	¥¥¥
R17	Communication	15 years +	One-star	Chengdu	Sichuan	¥¥

* Price: “¥¥” means a moderate spend; “¥¥¥” means special occasion; “¥¥¥¥” means spare no expense (Sourced from the Michelin Guide).

**Table 3:** Summary of analysis results.

Local Community	Local Knowledge	Translocality
➢ **Local suppliers** High-quality ingredients Timeliness of the supply chain Establishing trust in the supply chain Increasing the ingredient’s demands Supply chain stability ➢ **Local customers** Support from local customers Satisfying the local market Communicating with customers Being loyal to the local flavour Prioritising local customers ➢ **Local teams** Establishing a longstanding local team Local sponsorships Considering the team members’ needs Taking social responsibility	➢ **Products** Understanding the local produce Distinguishing the ingredients’ quality Food grown by the restaurants ➢ **Recipes** Following seasonality Conserving culinary heritage Improving traditional recipes ➢ **Cooking techniques** Applying various cooking techniques to cook local food Mastery of traditional cooking techniques Conservation of traditional cooking techniques Demonstrating cooking techniques to customers ➢ **Local culture** Designing tableware Decorating with local ornaments Demonstrating local culinary culture Naming by local specialities	➢ **Menu formulation** Combining indigenous cooking techniques and local products Adhering to tradition while innovating ➢ **Introducing indigeneity** Emerging locality and indigeneityTranslating the dishes’ names accurately Gaining word-of-mouth from local customers

## Data Availability

The original contributions presented in the study are included in the article, further inquiries can be directed to the corresponding author.
